# Ectopic Pregnancy in Uncommon Implantation Sites: Intramural Pregnancy and Rudimentary Horn Pregnancy

**DOI:** 10.1155/2015/536498

**Published:** 2015-12-24

**Authors:** Yi Wang, Fan Yu, Li-Qin Zeng

**Affiliations:** Department of Gynaecology, Guangdong Women and Children Hospital, Guangzhou 510010, China

## Abstract

Ectopic pregnancy is commonly located in the fallopian tube. Nevertheless, two unusual types of ectopic pregnancy, intramural pregnancy and rudimentary horn pregnancy, seriously threaten maternal life. The diagnosis and treatment of these unusual implantation sites present a clinical challenge. In this study, we illustrated the two unusual types of ectopic pregnancy and summarized the current data regarding diagnosis and optimal treatment from our experience.

## 1. Case Series

### 1.1. Case One

**Figure 1:**
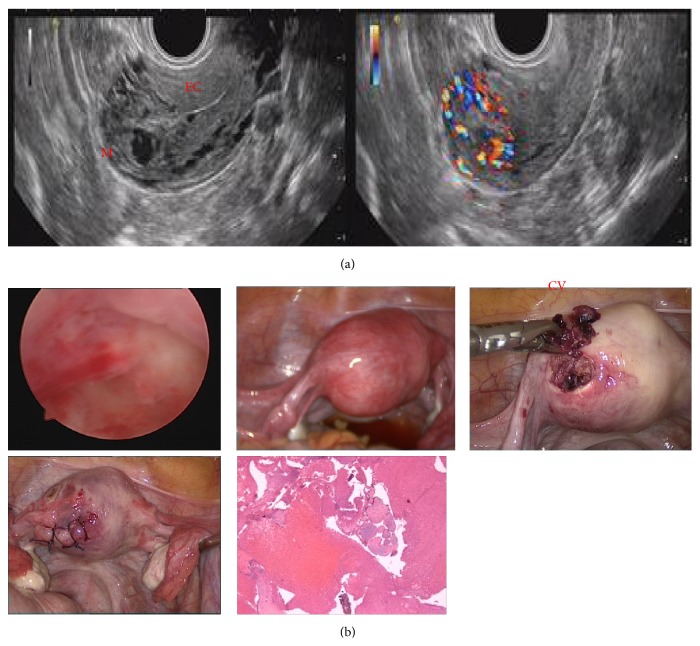
(a) Color Doppler transvaginal sonography of the transverse view of the uterus showing an endometrial cavity (EC) and mass (M). (b) Hysteroscopy reveals an empty uterine cavity without gestational tissue. Laparoscopic findings showing an intramural pregnancy near the left fundus. Histological specimens showed that chorionic villi (CV) were identified in the intramural pregnancy.

A 21-year-old female, G3P1A1, presented to our hospital because ultrasound scan revealed intrauterine tissue residues after two curettage operations. In another hospital, two months ago, she was initially misdiagnosed with a missed abortion at approximately 8 weeks of gestation and then suction curettage was performed. However, she suffered persistent vaginal bleeding after the first curettage and ultrasound showed intrauterine tissue residues. Therefore, the second curettage was carried out. However, the biopsy specimen was not obtained in the two curettage operations. In our hospital, transvaginal sonography revealed an ill-defined fundal mass (30 mm × 25 mm) near the left fundus. Color-flow Doppler analysis revealed high blood flow at the periphery of the fundal mass, and it was difficult to differentiate the boundary from the myometrium or endometrial cavity (Figure 1(a)). Her serumb-hCG level was 1069 mIU/mL.

We suspected an embryonic left cornual pregnancy or an invasive molar pregnancy and therefore decided to perform hysteroscopy and laparoscopy. The initial hysteroscopy revealed an empty uterine cavity with endometrial thinness and visible bilateral ostia, which negated the possibility of cornual pregnancy (Figure 1(b)). Laparoscopy revealed that the uterine size was a little bigger with an unruptured mass (2 cm in diameter) which protruded from the left fundal myometrium and was distinct from both fallopian tubes (Figure 1(b)). Both the ovaries and the fallopian tubes appeared normal. After the diagnosis of intramural pregnancy, terlipressin 6U was injected into the myometrium around the base of the mass. Surgery was performed and an incision was made in the uterine serosa, forcing the extrusion of gestational tissue. Then we found that the tissue was implanted in the myometrium without a connection to the endometrial cavity or fallopian tubes. Then the wound was repaired with number 1 Monocryl sutures (Figure 1(b)). Then the patient's postoperative condition was stable and serumb-hCG level declined to the negative value in the next 40 days. Microscopic examination of the excised tissue confirmed villi without an obvious molar pattern.

### 1.2. Case Two

**Figure 2:**
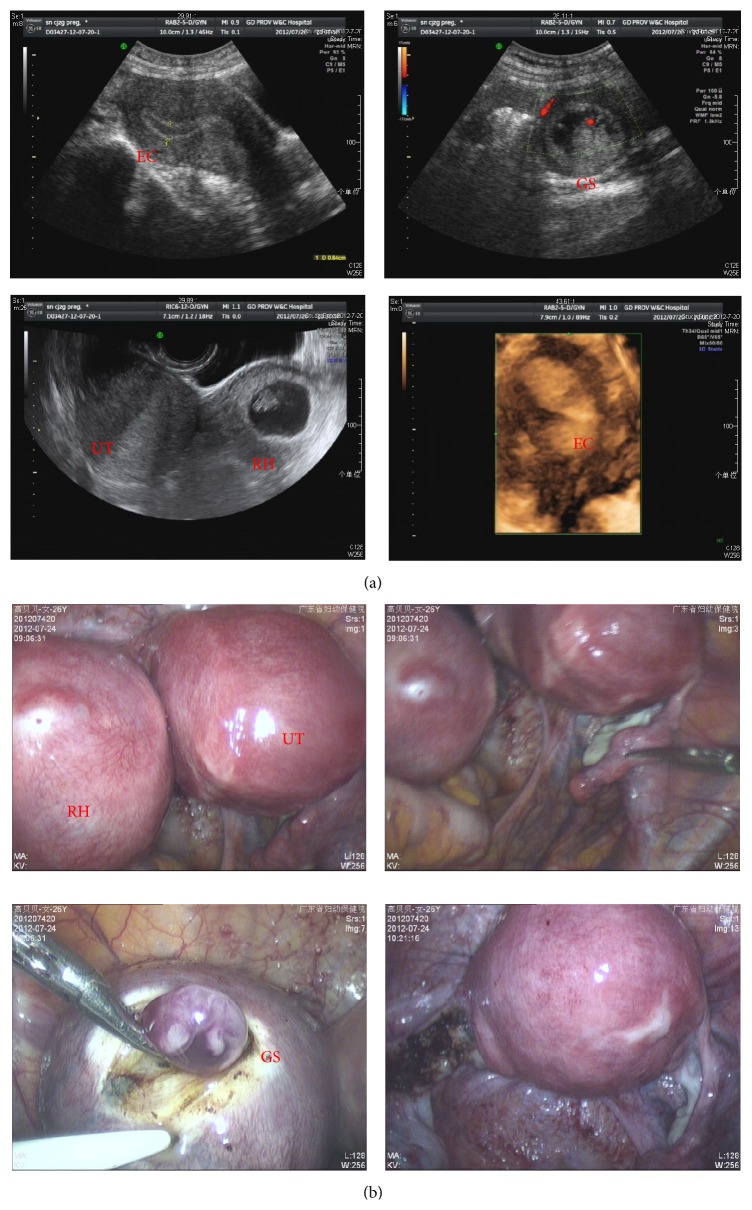
(a) Ultrasound revealed a right unicornuate uterus (UT) with an endometrial cavity (EC) and a noncommunicating rudimentary horn (RH) with a gestational sac (GS). There is an embryo (E) in the gestational sac with visible fetal heart beat. (b) Laparoscopy findings showing a left noncommunicating rudimentary uterine horn attached to a normal right horn.

A 26-year-old female, G1P0, presented to the emergency department at her 53rd day of amenorrhea with abdominal pain. She had normal menstrual periods without the history of dysmenorrhoea. At admission, the patient's general condition was good and her vital signs were normal. Pelvic examination revealed a single cervix with normal uterus on the right side. On the left side, a soft painful mass (60 × 60 mm) was palpable. An ultrasound investigation was performed, including an initial two-dimensional (2D) ultrasound assessment of her pelvis with the selection of the region of interest and the acquisition of a three-dimensional (3D) ultrasound. The investigation revealed a right unicornuate uterus with the size of 66 mm × 42 mm × 40 mm and an endometrial thickness of 8 mm. Lining her left ovary, a noncommunicating rudimentary horn with a gestational sac of 33 × 20 mm was described. There was an 11 mm long embryo in the gestational sac with visible fetal heart beat. Both ovaries were normal in terms of morphology and volume (Figure 2(a)). Bilateral kidneys were normal.

The patient opted for laparoscopic surgery. Laparoscopy confirmed a left noncommunicating rudimentary uterine horn attached to a normal right horn. The wall of rudimentary horn was thin. Bilateral tubes and ovaries were normal. The rudimentary horn was excised with the ipsilateral fallopian tube (Figure 2(b)). Left ovary was left. Patient had an uneventful postoperative recovery and was authorized to leave in 4 days.

### 1.3. Case Three

A 27-year-old G1P0 woman at 15+^6^ weeks of amenorrhea presented with severe abdominal pain 2 days before admission. At arrival, she was noted to be in hypovolemic shock with pallor, hypotension (88/47 mmHg), and tachycardia. The abdomen was tense with the symphysis-fundal height of 18 weeks. No vaginal bleeding was noted. She had abdominal pain 7 days ago, but she was told that the pregnancy was normal in another hospital. No other significant histories were noted. Further assessment with transabdominal ultrasound showed a viable fetus with the 16-week size beside the normal uterus. Much abdominal effusion was described. Pregnancy in a rudimentary horn of the uterus was suspected with a differential diagnosis of an abdominal pregnancy. Then 3 mL of nonclotting blood was pumped by abdominal paracentesis.

**Figure 3:**
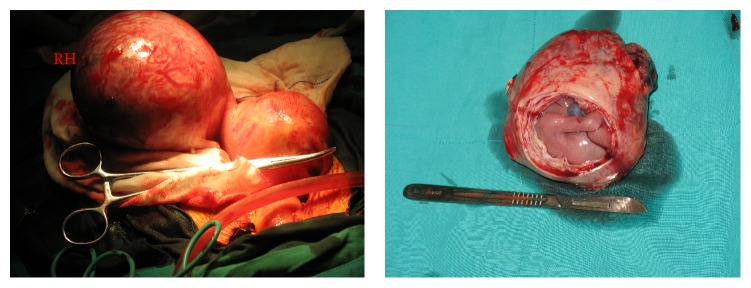
Intraoperative findings revealed a unicornuate uterus with left rudimentary horn pregnancy.

An emergency laparotomy was performed immediately. Intraoperative findings revealed a unicornuate uterus with left rudimentary horn pregnancy (Figure 3). The left rudimentary horn was not ruptured but covered with much engorged blood vessel. It was observed that the horn was not connected to the contralateral uterine cavity. Both the ovaries and fallopian tubes were normal. The left horn and tube were removed together with the fetus. A total volume of 1.5 L of hemoperitoneum blood was removed intraoperatively. She was discharged in a healthy condition on the 8th postoperation day after proper contraceptive advice.

## 2. Discussion

### 2.1. Intramural Pregnancy

Intramural pregnancy is one of the rare types of ectopic pregnancy. It refers to a uterine conceptus within the myometrium, without the connection with the fallopian tubes or endometrial cavity. This type of pregnancy accounts for less than 1% of all ectopic pregnancy [1]. It was first reported by Doederlein and Herzog in 1913 [2]. Complications resulting from intramural pregnancy include inevitable uterine rupture with resultant hemorrhage. The reported longest gestation with fetal survival is 30 weeks but with resulting cesarean hysterectomy because of uterine rupture [3]. The cause of intramural pregnancy is unclear, and the possible risk factors include a prior uterine trauma, adenomyosis, pelvic surgery, and in vitro fertilization [4]. Uterine traumas result in a sinus tract within the endometrium and may be the consequence of cesarean section, myomectomy, dilation, and curettage [4].

It is difficult to preoperatively diagnose the intramural pregnancy, which is often misdiagnosed. Diagnostic modalities may include ultrasound, a computed tomography (CT) scan, and magnetic resonance imaging. The diagnosis of intramural pregnancy requires clear visualization of the endometrial-myometrial junction in order to delineate the endometrial cavity and detect extension of trophoblast into the myometrium. According to the ultrasound characteristics, Luo et al. divided the intramural pregnancy into three types: gestational cyst type, mass type, and uterine rupture type. The gestational cyst type or mass type presented with an empty uterus and a gestational sac or mixed mass in the myometrium, which can be separated from the endometrium. The sac or mass did not communicate with the uterine cavity. Abundant blood flow was observed around the sac or in the mass, while massive hemoperitoneum was found in the uterine rupture type [5]. Compared with MRI, trophoblast tissue shows a typical tree-like structure, which may be conducive to diagnosis [6]. In the cases where clinical diagnosis is unclear, hysteroscopy may be a minimally invasive approach to exclude corneal ectopic pregnancy. In case one, we used hysteroscopy to assist the diagnosis and clearly defined the relationship between the mass and the endometrial cavity.

The management of intramural pregnancy depends on the size of the lesion, patient status, and also the desire for future fertility. Previous treatment modalities for intramural pregnancy include expectant management, surgical enucleation, uterine artery embolization, systemic or local methotrexate administration, and hysterectomy [7]. Because only 51 cases have been reported till 2012 [8], single universal treatment method for intramural ectopic pregnancy is not available. In the case presented here, we successfully managed an intramural pregnancy with hysteroscopy and laparoscopy.

### 2.2. Rudimentary Horn Pregnancy

Rudimentary horn pregnancy (RHP), described in cases  2 and 3, occurs once in 76000 pregnancies. In 70% ~ 90% of cases, the horn is noncommunicating. Ultrasound scanning is only 29% sensitive to diagnose RHP. Most RHP cases are still diagnosed during surgery [9]. The first RHP was described by Vassal and Mauriceau in 1669. Rudimentary horn pregnancy results in the rupture of the horn in 80% ~ 90% of cases by second trimester, and only about 10% of full term pregnancy.

Rudimentary horn pregnancy represents a very serious life-threatening pathological condition. Though the maternal mortality rate has been reduced from 18% in the nineteenth century to 0.5% now, the importance of early diagnosis to prevent morbidity and mortality cannot be overemphasized [10]. The differential diagnosis includes a tubal, corneal, or intrauterine pregnancy in a bicornuate uterus. Ultrasound seems to be the most helpful and practical radiologic investigation. Continuity of the cervix only with the nonpregnant uterine horn, the absence of continuity of gestational mass with the cervix observed on transvaginal sonography (TVS), was the important imaging findings [10]. Extensive diagnostic techniques, including MRI and hysteroscopy, may be required to differentiate accurately RHP from a pregnancy in a bicornuate uterus. The continuity between the endometrium lining the gestational sac and the other uterine horn is typical for a pregnancy in a bicornuate uterus. In case of persistent doubt, hysteroscopy can easily determine the absence of a cervical channel to the uterine horn and consequently discriminate a rudimentary horn from a bicornuate uterus [11].

The surgery of excising the rudimentary horn and ipsilateral fallopian tube is the main treatment method of rudimentary horn pregnancy. When the rudimentary horn pregnancy is small and facilities are available, it may be possible to resect it by laparoscopy. Others described the administration of methotrexate for termination of an early pregnancy in a rudimentary horn followed by elective laparoscopic resection [12]. In both cases of rudimentary horn pregnancy in the paper, we succeeded to excise the rudimentary horn and ipsilateral fallopian tube.

## 3. Conclusion

Two unusual types of ectopic pregnancy (intramural pregnancy and rudimentary horn pregnancy) were illustrated and discussed in the paper. Ectopic pregnancy at the unusual location is encountered much less frequently but is more morbid. The treatment of these types of unusual ectopic pregnancy may not be as commonplace as the treatment of tubal pregnancy. However, through early diagnosis and effective planning, the treatments can be equally effective.
